# Acute myeloid leukemia with gastric carcinoma: A case report of a double malignancy

**DOI:** 10.1097/MD.0000000000039285

**Published:** 2024-08-09

**Authors:** Qiuxia Wan, Yongzhen Chen, Suyun Wang

**Affiliations:** a Department of Hematology, Shenzhen Longhua District Central Hospital, Shenzhen, Guangdong China; b Department of Neurology, Shenzhen Longhua District Central Hospital, Shenzhen, Guangdong China.

**Keywords:** acute myeloid leukemia, adenocarcinoma of the cardia, gastric carcinoma, multiple primary cancer

## Abstract

**Rationale:**

Multiple primary cancers (MPC) are malignant tumors that manifest as multiple primary tumors diagnosed in the same patient, either simultaneously or sequentially. Billroth first proposed the concept in 1889. Here, we report a rare case of untreated acute myeloid leukemia (AML) and adenocarcinoma of the cardia.

**Patient concerns:**

A 58-year-old male with muscle and joint pain for >1 month was admitted to the hospital with severe chest pain for 3 hours on July 14, 2023. The patient had chest tightness, shortness of breath, and dyspnea. The skin, mucosa, and lips of the patient were slightly pale and the sternum had mild tenderness. Other systemic examinations did not reveal any obvious abnormalities. The results of routine blood tests on admission were as follows: white blood cells, 7.46 × 10^9^/L; red blood cells, 2.32 × 10^12^/L; hemoglobin, 90 g/L; and platelets, 62 × 10^9^/L.

**Diagnosis:**

The patient was diagnosed with acute myeloid leukemia (FLT3, DNMT3A, U2AF1, and SMC3 mutations; KMT2A amplification; high-risk) and adenocarcinoma of the cardia.

**Interventions:**

The patient was treated with azacitidine + Veneckla chemotherapy, and through precise regulation, the patient survived the period of bone marrow suppression. He was unable to achieve complete relief and finally underwent allogeneic hematopoietic stem cell transplantation in February 2024.

**Outcomes:**

Bone marrow cytology and minimal residual disease analysis indicated complete relief on April 22, 2024, with the bone marrow exhibiting complete chimerism (99.63%). The patient and his family members decided to seize the opportunity to perform radical surgical treatment for gastric cancer on May 16, 2024.

**Lessons:**

The development of medicine, especially in oncology, has led to an increased possibility of developing multiple cancers. Clinically, some doctors may not be aware of the existence of multiple primary cancers, especially simultaneous carcinomas, which can be easily missed or misdiagnosed.

## 1. Introduction

Multiple primary cancers (MPC) are malignant tumors that manifest as more than 1 primary tumor in the same patient, either simultaneously or sequentially.^[1]^ The development of medicine, especially in oncology, has helped prolong cancer patient survival but has also led to an increased possibility of developing multiple cancers. There have been many reports in the literature on MPCs involving solid organs; however, a double malignancy involving the hematopoietic system and a solid organ is less common, most likely developing secondary leukemia after chemotherapy and radiotherapy.^[2]^ Here, we report a rare case of untreated acute myeloid leukemia (AML) and adenocarcinoma of the cardia.

## 2. Case presentation

A 58-year-old male with muscle and joint pain for more than 1 month was admitted to the hospital with severe chest pain for 3 hours on July 14, 2023. The patient had chronic muscle and joint pain and sudden severe chest pain accompanied by chest tightness, shortness of breath, and dyspnea, but no nausea, vomiting, acid reflux, bloody stool, or other discomfort. The patient had been diagnosed with diabetes 10 years ago and was currently treated with oral gliclazide with good glycemic control. The patient had a smoking history of more than 20 years, with approximately 15 cigarettes smoked per day. There had no history of excessive alcohol use. The patient denied any family history of cancer. On admission, the results of the physical examination were as follows: temperature, 36.5°C; pulse, 84 beats/min; breathing frequency, 33 breaths/min; and blood pressure, 116/79 mm Hg. The skin, mucosa, and lips of the patient were slightly pale and the sternum had mild tenderness. Other systemic examinations did not reveal any obvious abnormalities. The results of routine blood tests on admission were as follows: white blood cells, 7.46 × 10^9^/L; red blood cells, 2.32 × 10^12^/L; hemoglobin, 90 g/L; and platelets, 62 × 10^9^/L. The blood cell morphological examination results showed that the percentage of lymphocytes was 14%, the percentage of monocytes was 14%, the percentage of primitive cells was 3%, the percentage of late granulocytes was 1%, and the percentage of primitive blood cells was 3%. The laboratory work-up showed folic acid, 6.11 ng/mL; vitamin B12, 805.3 pg/mL; and ferritin (1794 ng/mL). The results of the antibody tests were as follows: platelet-related antibody IgA, 0.66%; IgG, 1.52%; IgM, 0.10%; D-dimer, 5.41 μg/mL; alpha-fetoprotein, 1.24 ng/mL; carcinoembryonic antigen, 7.030 ↑ng/mL; cancer antigen 19 to 9, 10.53 U/mL; and HbA1c, 7.80%. No significant abnormalities were observed in cardiac enzymes, troponin I, BNP, electrolytes, liver function, or renal function. The patient had normal ECG results. Cardiac color ultrasonography revealed no structural abnormalities. Cranial and chest computed tomography (CT) showed no brain lesions, chronic inflammation in the lower lobe of the left lung, or multiple pulmonary alveoli in the left lung. Coronary computed tomography angiography (CTA) + pulmonary artery CTA + aortic CTA showed no significant abnormalities on coronary artery CTA, no significant abnormalities on pulmonary artery CTA, calcified plaque of the aortic arch wall, soft plaque in the thoracic aorta, and mild stenosis of the lumen.

We realized the seriousness of the patient’s condition and took the time to perform a series of necessary tests. Examination of bone marrow cytology showed that the proportion of the granulocyte system increased significantly; primitive granulocytes consisted of 14% nuclear cells, and POX-positive and abnormal granulocytes comprised 8% of nuclear cells. Flow cytometry of bone marrow cells showed that 7.5% of myeloid primitive cells had abnormal immunophenotypes. Chromosomal karyotype analysis of the bone marrow samples showed no abnormalities. Screening for common fusion genes in myeloid leukemia yielded negative results. The comprehensive detection results of MDS-related genes were as follows: FLT3 gene FLT3-ITD frame insertion/repetition mutation (mutation abundance 4.87%), U2AF1 gene p.S34F hot spot mutation (mutation abundance 35%), DNMT3A gene p.R882H hot spot mutation (mutation abundance 34.4%), and SMC3 p.R381Q missense mutation (mutation abundance 32.2%), and an increased number of copies of the KMT2A gene (chr11q23.3) in some regions (exons 2–8, approximately 13.9 kb in length; >2 copies).

**Figure 1.**
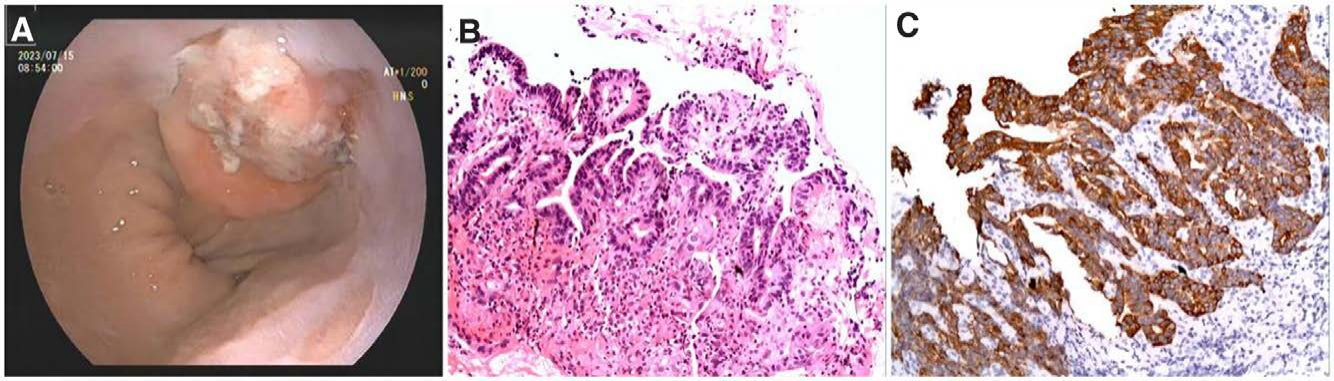
(A) The gastrointestinal endoscopy showed a cardiac mass. (B, C) Pathological diagnosis: glandular cell dysplasia and infiltration of inflammatory cells (HE, ×400). Cytokeratin was positive. The morphological and immunohistochemical diagnosis were consistent with medium-low differentiated adenocarcinoma.

The results of digestive endoscopy were as follows: cardiac mass; hyperemia and edema of the antrum and chronic gastritis. Pathological diagnosis: (mucosa of the cardia) medium-low differentiated adenocarcinoma. The immunohistochemical results were as follows: CD117 (small amount +), CD15 (+), CD20 (B-cell +), CD3 (T-cell +), CD34 (blood vessel +), CD56 (partial +), CK (+), desmin (-), KI-67 (+, 70%), MPO (+), MUC5AC (partial +), P53 (+, wild-type), CD45 (+), and SM (partial +). Unfortunately, the patient was diagnosed with adenocarcinoma of the cardia (Fig. 1).

**Figure 2.**
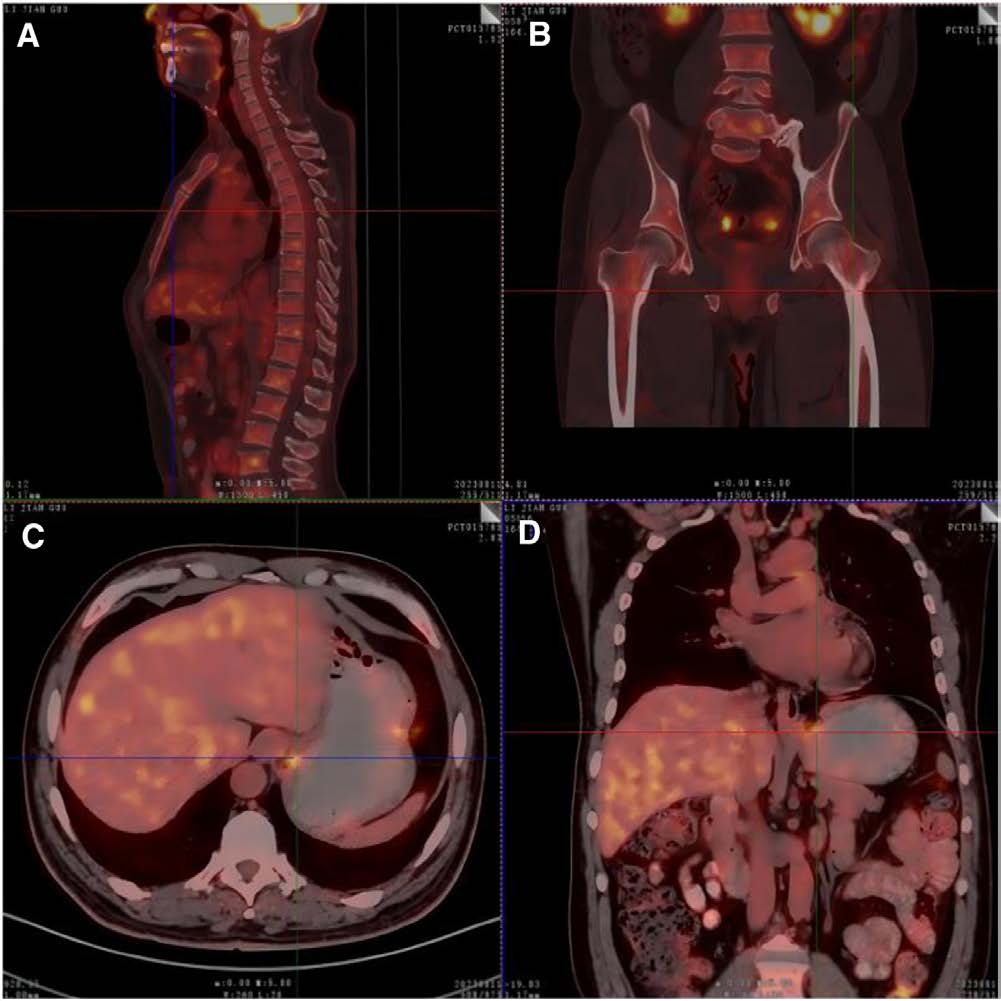
(A–D) PET-CT examination showed diffuse and uneven hypermetabolism of systemic bone marrow, accompanied by uneven bone marrow density. An irregular mass at the gastroesophageal junction, accompanied by thickening and increased metabolism. PET-CT = positron emission tomography-computed tomography.

The results of the PET/CT examination were as follows: Diffuse and uneven hypermetabolism of the systemic bone marrow is accompanied by uneven bone marrow density, which is considered a blood-derived malignant tumor lesion (acute leukemia). An irregular mass at the gastroesophageal junction, accompanied by thickening and stiffness of the tube wall, a narrow lumen, and increased metabolism, was diagnosed as cardiac cancer, and the boundary between the tumor and tissue around the esophageal hiatus was not clear (Fig. 2).

In the present case, the final diagnosis was acute myeloid leukemia (FLT3, DNMT3A, U2AF1, and SMC3 mutations; KMT2A amplification; high-risk) with adenocarcinoma of the cardia. The patient was treated with azacitidine and venetoclax. During chemotherapy, the patient experienced myelosuppression and received transfusions of red blood cells and platelets. The patient survived the period of bone marrow suppression. He was unable to achieve complete relief and finally underwent allogeneic hematopoietic stem cell transplantation in February 2024. Bone marrow cytology and minimal residual disease analysis indicated complete relief on April 22, 2024, with the bone marrow exhibiting complete chimerism (99.63%). The results of the blood routine examination exhibited a gradual improvement. Combined with the consultation opinions of general surgeons, the patient and his family members decided to seize the opportunity to perform radical surgical treatment for gastric cancer on May 16, 2024. We will continue to pay attention to the effects of subsequent treatments on patients.

## 3. Discussion

MPCs occurring in the same patient has been previously reported in the literature. This concept was first proposed by Billroth in 1889, and its incidence ranged from 3.16% to 7.49%.^[3]^ In 1932, Warren first established certain criteria to diagnose 2 separate malignant neoplasms^[4]^: each tumor must be clearly malignant, each tumor must be geographically separate and distinct, and the possibility that the second tumor represents metastasis should be excluded from a diagnostic study. Multiple primary cancers can be divided into 2 types: simultaneous cancer and heterochronous cancer, with intervals of less than 6 months for synchronous cancer and more than 6 months for heterochronous cancer.^[3]^

Our case was clearly simultaneous carcinoma. Solid organs commonly involved in MPC include the stomach, colon, breast, and esophagus.^[5]^ Hematological malignancies coexisting with other primary tumors in MPC usually include multiple myeloma, myelodysplastic syndrome, non-Hodgkin lymphoma, and chronic myelogenous leukemia.^[6,7]^ Clinically, repeat carcinomas with leukemia and other malignant tumors have been found,^[8]^ most of which are chronic leukemia. Untreated acute myeloid leukemia with synchronous repeat carcinoma of gastric cancer is rare.

Acute myeloid leukemia (AML) and gastric cancer are 2 completely different tumor types. Leukemia is a hematological malignancy caused by malignant clonal proliferation of hematopoietic stem cells. AML cells are mostly primitive and early naive cells. The disease progresses rapidly with a natural course of only a few months. Gastric cancer (GC) is a malignancy of the digestive system that originates in the gastric mucosa. Under the influence of various factors, such as poor living environment, unhealthy diet, HP infection, and genetic factors, it gradually progresses to gastric cancer after chronic inflammation and dysplasia. In the present case, the patient was treated for the chest pain. After improvements in routine blood tests, peripheral blood cell morphology, bone marrow cell morphology, flow cytology, gastroscopy, gastric tissue pathology, and other related examinations were determined to indicate both acute myeloid leukemia and gastric cancer. PET/CT results excluded the possibility of metastasis to the same cancer. According to the relevant definition, this case was an acute myeloid leukemia associated with gastric cancer.

The etiology of multiple primary malignant tumors is complex and includes environmental factors, genetic predisposition, immunological impairment, previous medical treatment, sex, and hormonal factors.^[9,10]^ It is now widely accepted that cigarette smoking is a common risk factor for the development of acute leukemia and gastric cancer.^[11]^ This patient was a middle-aged male with a history of smoking, Helicobacter pylori infection, no family history of tumor, and no radiotherapy or chemotherapy and was considered to be related to host susceptibility, body immune deficiency, smoking, etc.

Chemotherapy and hematopoietic stem cell transplantation are the primary treatment modalities for acute myeloid leukemia. Surgery remains the main treatment for gastric cancer, and radiotherapy and chemotherapy are helpful in improving the cure rate of gastric cancer. The long-term survival of patients with leukemia is generally the goal when leukemia is combined with solid malignant tumors. Chemotherapy alone has no effect on gastric cancer in patients with acute leukemia complicated by gastric cancer. At present, it is recommended to administer chemotherapy for acute leukemia first and then undergo radical surgical surgery for gastric cancer after complete remission.^[12]^ However, after gastric cancer surgery, patients may develop serious complications such as infection, respiratory failure, thrombocytopenia, and coagulopathy. This may be related to the coexistence of acute leukemia and the use of preoperative chemotherapeutic drugs. The incidence of leukemia combined with solid tumors is low. Diagnoses and treatments for different patients can vary significantly, necessitating a comprehensive evaluation of factors including tumor malignancy, treatment effectiveness, treatment indications, and patients’ general health conditions. Based on limited literature, there is currently no standard treatment strategy. In clinical practice, given the rapid progression of symptoms and signs in acute leukemia, we prioritize treatment for leukemia or administer combination chemotherapy to suitable patients.^[13]^ In fact, many patients with dual malignant tumors, including leukemia and solid organs, do not receive effective treatment.

The patient was diagnosed with acute myeloid leukemia complicated by cardia cancer in a timely manner, and reasonable treatment methods such as leukemia chemotherapy, bone marrow transplantation, and gastric cancer radical surgery were adopted to achieve a favorable prognosis. Therefore, early diagnosis and treatment as well as interdisciplinary cooperation are essential. The prognosis of these patients largely depends on the malignancy and stage of the dual tumors at the time of diagnosis, as well as the patient’s age and overall health.^[14]^ The prognosis of malignant tumors combined with acute leukemia is very poor, generally only a few months to 1 year of survival.

With advances in modern medical technology and enhanced cancer treatment options, many patients with cancer have been cured, leading to longer survival periods. It is only then that subsequent cancers have the chance to manifest, and the detection rate of multiple primary malignant neoplasms is gradually increasing. The diagnosis of acute leukemia combined with solid tumors requires case-by-case analysis. Diagnosing solid tumors combined with acute leukemia is relatively straightforward, as these patients often exhibit obvious abnormalities in their blood, making concurrent hematological tumors more likely to be considered in clinical practice.^[15]^ Diagnosing acute leukemia combined with solid tumors is challenging given the early stages of atypical clinical symptoms, which can affect multiple organs or sites across the body. As not all affected areas can be pathologically examined at the initial diagnosis, there is a high risk of missed diagnoses when these diseases coexist. Therefore, if individual treatments are ineffective or if patterns do not conform to tumor metastasis patterns, 1 should be vigilant for the presence of MPCs during solid tumor treatment. Positron emission tomography-computed tomography scans have significant diagnostic value; hence, they are advised for acute leukemia and solid tumor patients before and after treatment whenever possible.^[16]^ PET/CT plays an important role in tumor staging in patients with chronic lymphocytic leukemia/small lymphocytic leukemia.^[17]^ It is also helpful in detecting multiple coexisting cancers.^[18]^ Combining auxiliary research, such as immunohistochemistry and flow cytometry, is often essential for achieving an accurate and comprehensive diagnosis and staging of tumors.

Clinically, some doctors believe that a patient can only have 1 cancer or that the same organ can only have cancer in 1 place and are not aware of the existence of multiple primary cancers, making it easy to miss a diagnosis or misdiagnosis. The clinical presentation of a second primary cancer is often confused with the first cancer or masked by its clinical symptoms of the first cancer. Metachronous second primary carcinoma mostly occurs 1 to 3 years after the first surgical carcinoma,^[19]^ which coincides with the time of recurrence or metastasis of the first cancer. At diagnosis, only cancer recurrence or metastasis is often considered regardless of the possibility of multiple primary cancers. After the first cancer was cured, a diagnosis of non-neoplastic disease was considered when recurrence or metastasis was excluded.

The patient was admitted with chest pain, dyspnea, and other discomfort. Routine blood examination revealed hemoglobin and thrombocytopenia, and CTA examination excluded cardiopulmonary vascular disease. We also need to have a high suspicion of digestive system diseases, understand the true nature of these diseases, and avoid missing diagnoses. We hope that this case report will attract the attention of clinicians in the future.

## 4. Conclusion

These 2 diseases occur in different organs and rarely in the same individual. However, whether they have any internal relevance is currently unknown and requires further investigation. There are very few reports on the association between these 2 diseases. We suspect that this combination may have been caused by malignant transformation of upstream histiocytes and stem cells. It is generally believed that the treatment effect of multiple primary cancers is better than that of cancer recurrence or metastasis and that the probability of cure is greater. Similar to single primary cancers, as long as radical measures are taken, the prognosis remains good. The key problem is that clinicians have the correct understanding and vigilance of multiple primary cancers, so that they can take the correct treatment plan as soon as possible.

## Author contributions

**Conceptualization:** Qiuxia Wan, Yongzhen Chen.

**Data curation:** Qiuxia Wan, Yongzhen Chen.

**Formal analysis:** Qiuxia Wan, Yongzhen Chen.

**Investigation:** Qiuxia Wan, Yongzhen Chen.

**Methodology:** Suyun Wang.

**Supervision:** Suyun Wang.

**Writing – original draft:** Qiuxia Wan.

**Writing – review & editing:** Suyun Wang.
